# Endocrine disrupting potency of organic pollutant mixtures isolated from commercial fish oil evaluated in yeast-based bioassays

**DOI:** 10.1371/journal.pone.0197907

**Published:** 2018-05-22

**Authors:** Marek Łukasz Roszko, Marta Kamińska, Krystyna Szymczyk, Katarzyna Piasecka-Jóźwiak, Beata Chabłowska

**Affiliations:** 1 Department of Food Analysis, Institute of Agricultural and Food Biotechnology, Rakowiecka, Warsaw, Poland; 2 Department of Fermentation Technology, Institute of Agricultural and Food Biotechnology, Rakowiecka, Warsaw, Poland; Universite Clermont Auvergne, FRANCE

## Abstract

The aim of this work was to evaluate the activity of xenobiotic mixtures containing persistent organic pollutants isolated from commercial fish oil samples against sex hormone receptors, including estrogen and androgen. The applied bioassay was based on transgenic yeast strains. The mixtures were extracted from the samples using the semi-permeable membrane dialysis technique and analyzed with gas chromatography/ion trap mass spectrometry. It turned out that mixtures of chemicals isolated from fish oil may interact with human steroid sex hormone receptors in various ways: the tested samples showed both estrogenic and anti-androgenic activity. Calculated 17β-estradiol equivalents for the tested samples ranged between 0.003 and 0.073 pg g^–1^ (fat). Anti-androgenic activity expressed as the flutamide equivalent concentration was in the 18.58–216.21 ng g^–1^ (fat) range. Polychlorinated biphenyls and various DDT metabolites were the main fish oil pollutants influencing the receptors. Additivity and/or synergy between chemicals was observed in the ER/AR mediated response.

## Introduction

Endocrine disrupting chemicals (EDCs) are compounds that may interfere with human or wildlife endocrine systems and produce adverse developmental, reproductive, neurological and immune effects. A vast number of chemical compounds are known to mimic native human hormones like androgen (AR) and estrogen (ER) and to interact with their nuclear receptors (NRs) [1–3]. EDCs are able to interact with hormone NRs as agonists or antagonists [4]. They are able not only to activate the receptors in the absence of native hormones but also to increase or inhibit the response of endogenous ligands [5]. Therefore, environmental endocrine disrupters can be grouped into four categories: direct-acting agonists, direct-acting antagonists, indirect-acting agonists, and indirect-acting antagonists [6–7]. Competitive binding is a required condition for direct-acting compounds, and their affinity can be measured using ligand-binding assays. Some chemicals act indirectly, showing endocrine disrupting potency only after metabolic activation, i.e., after transformation into a related derivative but more native, structurally similar to hormone [8–9].

The presence of EDCs in the environment has raised concerns about the potentially harmful effects of exposure to such chemicals. The effects include modern civilization diseases such as cancer [10–12], and/or wildlife reproductive cycle disturbances [13–16]. The general public is chronically exposed to a wide group of EDCs from both natural and anthropogenic sources [17]. EDCs include polychlorinated biphenyls (PCBs), a number of pesticides, polycyclic aromatic hydrocarbons (after metabolic activation), brominated flame retardants, bisphenol A, pharmaceuticals, several classes of phytoestrogens and others; they are quite ubiquitous in the environment [18–23]. Several routes of exposure have been identified. However, food is the main exposure path of humans for lipophilic and persistent organic pollutants (POPs) [24]. Most commonly, EDCs in the food/environment are complex mixtures of different compositions, and therefore it is difficult to evaluate their biological potency by means of chemical analyses only. If any conclusions on hormonal activity of a mixed sample are to be meaningfully drawn from data on the potency/effectiveness of individual compounds of the mixture, several conditions must be met: (i) the mechanism of biological activity must be determinable; (ii) the measured activity of the compounds must be correlated with the induced biological effects; and (iii) the effects must be correlated with the combined ED potency. Unfortunately, such assumptions may be not true when ER- and/or AR-interacting compounds are evaluated due to the omnipresence of various compounds in the environment with both agonistic and antagonistic ED activity. In addition, such an approach is truly applicable only if the dose-response curve is linear. However, sigmoidal curves and non-monotonic responses are frequently observed for estrogenic/androgenic agents [2, 25–26]. Nevertheless, there is scientific evidence that chemical mixtures of substances sharing the same mode of action elicit predominately additive effects, both *in vitro* and *in vivo* [27–31]. In addition, synergistic effects could also be possible [32–33]. It should, however, be noticed that evaluation of interactions between chemicals is not an easy task, and wrong conclusions might be drawn due to a lack of information on the dose-response relationship [25,32].

The majority of the published studies on EDCs have focused on the ability of individual chemicals to interact with hormone receptors. Quite a few papers dealt with chemical mixtures derived from real-life food samples.

Fish oil is a food constituent. It is a rich source of omega-3 fatty acids and is commonly used as a dietary food supplement. Since fish oil might be contaminated with several classes of persistent chemicals, it has raised concerns on the potential health effects regarding its dietary use. These include potential endocrine disruption caused by the chemical mixtures present in these oils. As reported in our previous papers (e.g., [34]), fish liver oil may be heavily contaminated with chemicals of proven ED potency, including chlorinated biphenyls, brominated diphenyl ethers and its metabolites, or legacy organochlorine pesticides. In this work, estrogen/androgen agonistic and antagonistic activities of fish oil extracts were evaluated using optimized recombinant yeast assays. Since the combined ED potency of a mixture most likely depends on the NR binding potency of the individual mixture compounds, the isolated compounds were fractionated to identify the compounds mostly responsible for the observed activity.

## Materials and methods

### Chemicals/reagents

HPLC grade organic solvents were exclusively used in this study. N-hexane, cyclohexane, dichloromethane, and toluene were supplied by Sigma-Aldrich (Belefonte, PA, USA). Anhydrous sodium sulfate, potassium hydroxide, and sulfuric acid (96%) of p.a. grade were supplied by Avantor (Gliwice, Poland). Chlorophenol red galactopyranoside (CPRG), basic aluminum oxide, magnesium sulfate, copper sulfate, potassium phosphate, dihydrogen potassium phosphate, ammonium phosphate, iron (II) sulfate, L-threonine, L-leucine, L-histidine, adenine, L-arginine hydrochloride, L-arginine, L-methionine, L-tyrosine, L-isoleucine, L-lysine hydrochloride, L-phenylalanine, L-glutamic acid, L-valine, L-serine, thiamine, pyridoxine, pantothenic acid, inositol, biotin, aspartic acid and glucose were supplied by Sigma-Aldrich (Belefonte, PA, USA). Silica gel 60 (0.063–200 mm) was obtained from Merck (Darmstadt, Germany). YES/YAS yeast strains were obtained from Tigret (Warsaw, Poland). Pure PBDE standards IUPAC #1, 2, 3, 7, 8, 10, 11, 12, 13, 15, 17, 25, 28, 30, 32, 33, 35, 37, 47, 49, 66, 71, 75, 77, 85, 99, 100, 110, 116, 118, 119, 126, 138, 153, 154, 155, 166, 183, and 209 (>97%) were supplied by AccuStandard (New Haven, CT, USA). Aroclor PCB mixtures 1221, 1232, 1242, 1248, 1254, 1260, 1262, and 1268 were obtained from Sigma-Aldrich (Belefonte, PA, USA). o,p-DDT, p,p-DDE, p,p-DDD, p,p-DDT, heptachlor, o,p-DMDT, p,p-DMDT, HCB, trans-/cis-chlordan, and HCH were supplied by IPO (Warsaw, Poland). High purity (>97%) native PAH standards (naphthalene, acenaphtylene, acenaphtene, fluorene, phenanthren, anthracene, fluoranthrene, pyrene, benzo[c]fluorene, benzo[c]phenanthrene, chrysene, benzo[a]anthracene, cyclopenta[c,d]pyrene, benzo[b]fluoranthrene, benzo[j]fluoranthrene, benzo[k]fluoranthrene, 7,12-dimethylbenz[a]anthracene, benzo[a]pyrene, benzo[e]pyrene, 3-methylcholanthrene, indeno[1,2,3-cd]pyrene, dibenzo[a,h]anthracene, benzo[g,h,i]pyrelene, dibenzo[a,l]pyrene, dibenzo[a,i]pyrene, dibenzo[a,e]pyrene, dibenzo[a,h]pyrene, and 5-methylchryzene) were supplied by Dr. Ehrenstorfer (Augsburg, Germany) and Accustandard (New Haven, CT, USA). ER/AR native agonists 17β-estradiol and 17β-hydroxy-testosterone as well as antagonists flutamide and hydroxy-tamoxifen were supplied by Sigma-Aldrich (Belefonte, PA, USA). Streptomycin and ampicillin antibiotics were obtained from Sigma-Aldrich (Belefonte, PA, USA).

### Sample preparation

Our previously published sample preparation method with some major modifications was used. The fraction containing organic pollutants was isolated from fish oil samples (4 × 5 g) using semi-permeable dialysis [35]. Dialysates were rotary-evaporated to dryness and de-fatted with gel permeation chromatography. Separations were performed on a 500×10 (Omnifit, Cambridge, UK) column filled with Bio-Beads SX-3 styrene/divinylobenzene resin. The dichloromethane:cyclohexane (1:1) mobile phase eluted at a rate of 1 ml min^–1^, and a 2 ml sample loop was used. Fractions eluting between 25 and 55 ml were collected. Fractions eluted from individual sub-samples were combined, evaporated to almost dryness and re-dissolved in n-hexane. The phenolic fraction (containing hydroxylated polychlorinated biphenyls/brominated diphenyl ethers) was isolated through alkaline partitioning as described previously [36]. The neutral fraction was fractionated using a 3 g basic alumina column prepared as described previously [37]. The column was eluted with 35 ml of n-hexane, 35 ml of a dichloromethane:n-hexane mixture (1:99), and dichloromethane. The n-hexane fraction containing mainly aliphatic compounds was additionally passed through a glass column containing 1 g of acidic silica gel. Samples were evaporated to dryness and re-dissolved in 500 μl of ethyl acetate. To eliminate possible variations of fraction composition resulting from differences in recovery rates of individual compounds, one half volumes of each fraction were combined into a composite sample, while the second half volumes were analyzed individually. Then, 50 μl of each extract was transferred into a chromatographic vial for GCMS analysis, while the remaining portion was transferred into flat bottom glass chromatographic vials, evaporated to dryness within a gentle stream of nitrogen, and submitted for yeast-based bioassays. Statistical parameters of the applied analytical procedures were discussed in detail previously [35–37]. Recovery rates of the tested legacy pesticides from the spiked samples are shown in S1 Table.

### GC/MS analyses

Samples were analyzed in a TraceGC Ultra Thermo-Finnigan (Austin, TX, USA) gas chromatograph coupled to a PolarisQ Thermo-Finnigan (Austin, TX, USA) ion trap mass spectrometer equipped with a TriPlus autosampler (Austin, TX, USA) and a PTV injector. Chromatographic separations were performed using Rtx–17 MS medium-polarity fused-silica capillary column (Restek, Bellefonte, PA, USA) and a 5 m × 0.53 mm guard column/retention gap. The following temperature program was used during separations: 40°C 3 min hold, ramp to 200°C (25°C min^–1^), ramp to 250°C (1.4°C min^–1^), 2 min hold, ramp to 300°C (25°C min^–1^), 5 min hold. Then, 40 μl of the extract was introduced into the GC instrument using the solvent split injection method. Initial injector temperature was 40°C and the injection rate was 3 μl s^–1^. Helium split flow at the injection was set at 200 ml min^–1^. The split valve was closed after 1 minute and the temperature ramped to 300°C (25°C s^–1^) and held for 2 min. Subsequently, the split valve was opened, the flow rate set to 100 ml min^–1^, the temperature ramped to 320°C and held for 5 min. The mass spectrometer ion source was operated at 300°C and the GC transfer line temperature was set at 320°C. Other mass spectrometer operational parameters were as reported previously [36].

### Yeast estrogen and androgen bioassays

Yeast-based bioassays were performed according to Schulitz and Metzger [38] with some modifications. Minimal medium was prepared using 13.61 g KH_2_PO4, 1.98 g (NH_4_)_2_SO_4_, 4.2 g KOH, 0.2 g MgSO_4_, 0.8 mg Fe_2_(SO_4_)_3_, 50 mg L-leucine, 50 mg L-histidine, 50 mg adenine, 20 mg L-arginine, 20 mg L-methionine, 30 mg L-tyrosine, 30 mg L-isoleucine, 30 mg L-lysine, 25 mg L-phenylalanine, 100 mg L-glutaminic acid, 150 mg L-valine, and 375 mg L-serine dissolved in 1000 ml of deionized water. The solution was sterilized at 121°C for 15 min. A vitamin solution was prepared using 8 mg thiamine, 8 mg pyridoxine, 8 mg pantothenic acid, 40 mg inositol, and 0.4 mg biotin dissolved in 200 ml of deionized water and filtered through a sterile 0.2 mm nylon filter (Whatman PURADISC). The glucose solution (20%), aspartic acid solution (4 mg ml^–1^), threonine solution (24 mg ml^–1^) and 20 mM copper sulfate were sterilized at 121°C for 15 min. Combined growth medium was prepared by mixing solutions of glucose (5 ml), aspartic acid (1.25 ml), vitamins (1 ml), threonine (0.5 ml), copper sulfate (0.125 ml) and minimal medium (45 ml). Growth medium was spiked before use with ampicillin and streptomycin to give a final concentration of 1 mg ml^–1^ each.

Briefly, an overnight yeast (YES/YAS) culture with approx. 1.2 OD@690 nm was centrifuged at 1000 × g and re-suspended in 10 ml of fresh medium. Samples mixed with 120 μl of 4% ethanol solution in water were placed in 2 ml glass vials, inoculated with 60 μl of yeast suspension, shaken by hand, transferred into a tightly closed plastic box with the bottom covered with water (to assure sufficient humidity), and incubated for 8 hours at 36°C. Next, the samples were transferred into a 96-well plastic plate, and their optical density was measured. The culture was terminated with the addition of 20 μl of 0.1% Triton X–100 and 30 μl of lyticase (1 mg ml^–1^) and mercaptoethanol (50 mM) in 0.1 M phosphate buffer (pH 7.5) solution. Samples were incubated for 45 min at 25°C and afterwards mixed with CPRG solution (1.0 mg ml^–1^). Evolution of absorbance at 570 and 690 nm was measured six times at 30-minute intervals (total 3 hours).

### Test material

Fish (cod and shark liver) oils used as dietary supplements available through retail trade were used for the purposes of this study. Ten different oil brands were used. Pure standards of chemicals found at abundant concentrations in the oil samples including DDE, DDT, PCB mixtures, and several PBDE congeners were tested for estrogenic and androgenic activity. Chemicals were evaluated standalone, in combination and in the presence of a fixed concentration of native hormones (to evaluate antagonistic and combined effects).

### Data analysis

Xcalibur 1.2 (GCMS) and Galapagos 2.0 (Plate reader) software packages were used to acquire and analyze the data. The results are given as the means of at least two parallel determinations (±SD, if given). Authentic standards and mass spectra were used to identify chromatographic peaks. Compounds for which standards were not available were identified using mass spectra and quantified on the basis of the nearest available structural analogues. Calibration curves were plotted as peak area vs. analyte concentration.

If some cytotoxic effects inhibited yeast growth, the results of the respective bio-assay were excluded from the calculations. The samples were assessed in terms of agonist or antagonist activity against estrogen or androgen receptors. The results were expressed as mass equivalents of 17β-estradiol, hydroxy-tamoxifen (estrogen agonist), hydroxy-testosterone and flutamide (androgen agonist). Equivalents were calculated from EC 50 values estimated from dose–response curves plotted for samples and native hormones. In cases where EC 50 values were not achieved, EC 20 values were used for calculations. Estimations of response and/or activity for the tested chemicals mixtures were performed using two models: A–simple additive model $E_{mix}=\Sigma_{i=1}^{n}E_{i},$where E_mix_ is the estimated effect of a mixture of chemicals, and E_i_ is the effect of individual chemicals at a defined concentration; B–predictive model according to Payne et al. [27]$E_{mix}=\;E_{max}\left(1-\prod \left[1-F_{i}/E_{max}\right]\right)$, where E_max_ is the maximal effect (absorbance readings), and the F_i_ effect is predicted from the dose response curve of the individual chemical at a defined concentration. For the purpose of this study, experimental and measured values were used for calculations instead of using values estimated from the asymmetric Hill functions. Spearman’s rank test was used to test the correlation between the evaluated chemicals concentration and the hormone equivalents. One way ANNOVA was used to evaluate the significance of the differences between measured absorbance values and values estimated from the predictive models.

## Results and discussion

### Fish oil chemical contamination

**Fig 1:**
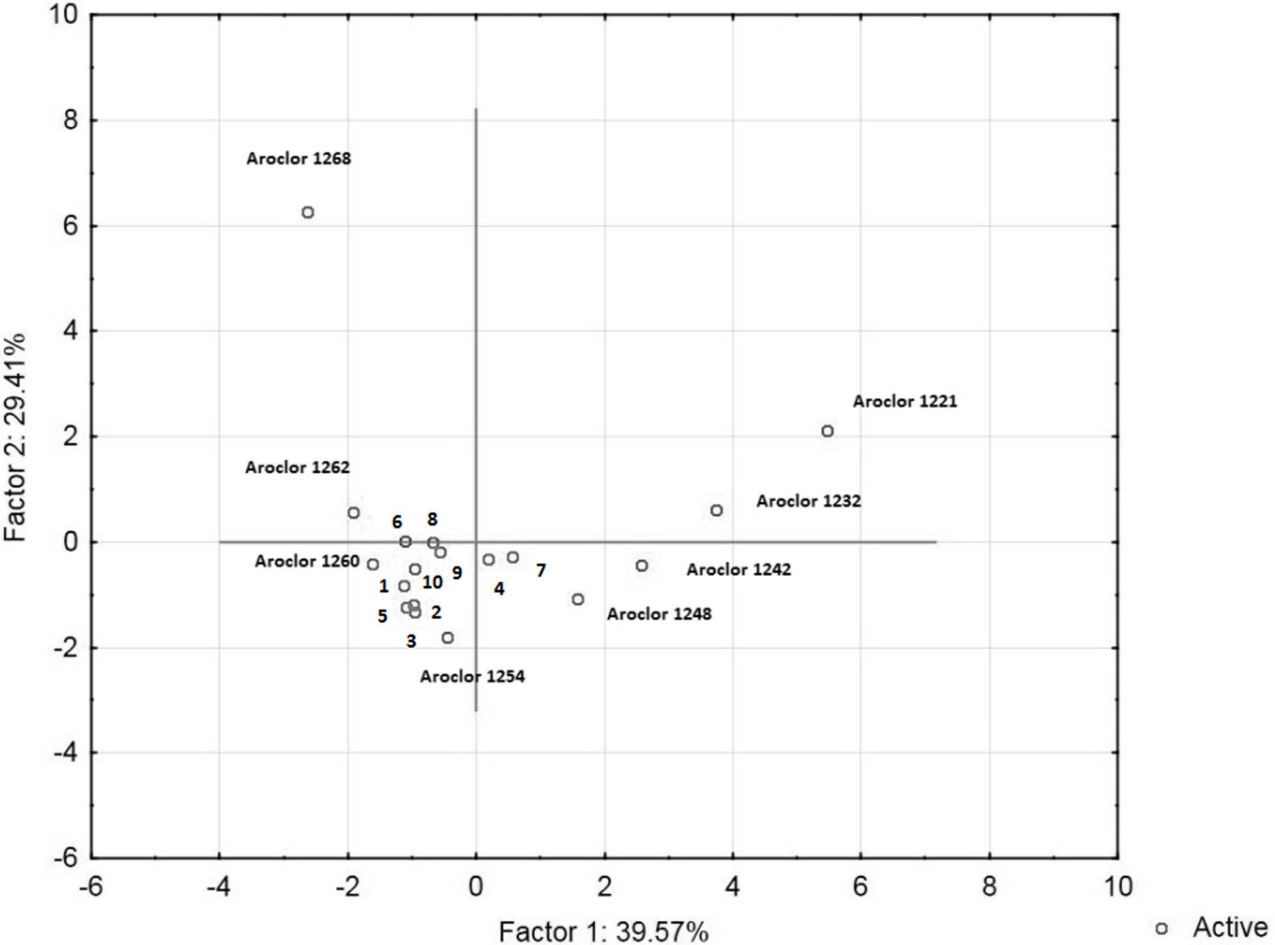
Principal component analysis scatter plot of PCB profiles found in the tested oil samples and in several PCB-based technical mixtures. Samples have been indicated with numbers.

POPs identified in the tested fish oil samples are listed in Table 1. Polychlorinated biphenyls (PCB) were found at the highest concentrations, consistent with our previous reports and the reports of other authors [34, 39–40]. Relatively high levels of various DDT metabolites and degradation products (including DDMU, DDD, and DDE) were also found, consistent with the reports of Rawn et al. [41], who reported high DDT concentrations in some types of fish oils. The Principal Component Analysis technique was used to compare profiles of PCB congeners found in the tested samples with the composition of some technical mixtures containing those compounds. The analysis showed that the found profiles were most congruent with profiles characteristic of the Aroclor 1260 technical mixture (Fig 1). However, since a number of both abiotic and biological factors affect the profiles, that does not necessarily mean that such a technical mixture was the real contamination source [34–35, 37, 42]. Nevertheless, to facilitate interpretation of the obtained results, PCBs concentrations were calculated as Aroclor 1260 equivalents using the authentic Aroclor technical mixture as the standard. Calculations were based on the summary chromatographic peak area of the most abundant congeners.

**Table 1:** Concentrations of organic pollutants identified in the tested oil samples.

	Sample [Mean ± SD] (n = 3)									
Compound	1	2	3	4	5	6	7	8	9	10
Concentrations of organic pollutants identified in the tested oil samples [ng g^–1^]										
Pentachlorobenzene	0.91±0.11	0.02±0.002	0.05±0.01	0.28±0.03	0.01±0.00	0.57±0.08	0.01±0.00	0.65±0.07	0.43±0.05	0.52±0.10
Hexachlorobenzene	103.07±13.14	0.54±0.09	0.03±0.00	0.31±0.04	2.16±0.34	58.67±6.60	3.51±0.48	74.33±12.41	4.87±0.57	43.71±7.28
Unident. chlorinated	92.84±17.75	1.50±0.21	3.25±0.40	43.67±8.57	7.77±0.79	53.44±6.77	12.65±2.07	71.07±13.89	76.04±14.21	61.09±6.82
Heptachlor	4.90±0.95	0.92±0.15	< 0.05	< 0.05	< 0.05	2.90±0.38	< 0.05	3.46±0.56	2.14±0.32	2.03±0.28
HCH-delta^1^	14.51±2.52	< 0.05	< 0.05	< 0.05	0.62±0.10	2.15±0.41	1.01±0.13	8.69±1.06	0.87±0.16	3.05±0.55
Octachlorostyrene	262.12±45.78	10.04±1.29	0.42±0.05	1.46±0.16	12.91±2.08	161.37±22.29	21.01±2.70	194.27±27.67	43.28±5.39	114.67±12.61
DDMU^2^	336.39±41.30	4.35±0.86	3.35±0.45	5.79±0.96	12.31±1.33	188.37±32.18	20.04±2.15	246.31±28.00	31.72±5.21	148.18±15.82
Chlordan	7.18±0.78	0.56±0.08	0.25±0.05	0.93±0.10	2.94±0.51	4.27±0.66	4.79±0.68	7.15±1.17	6.32±0.66	4.68±0.77
Trans-nonachlor	66.80±7.15	2.77±0.49	0.12±0.01	4.74±0.78	23.57±2.41	38.04±5.56	38.37±6.06	63.84±12.11	43.63±6.62	39.43±4.09
Trans chlordan	25.95±3.60	1.70±0.18	0.20±0.03	2.78±0.40	14.22±2.68	15.18±1.54	23.15±3.25	28.37±5.24	26.30±5.05	17.87±3.08
DDE^3^	1309±227.56	26.41±4.95	9.52±0.98	23.76±4.72	65.22±7.31	644.65±119.00	106.17±20.52	970.49±166.54	170.59±28.80	582.71±65.50
Nonachlor	12.07±1.62	0.68±0.10	0.05±0.01	1.94±0.38	7.55±0.98	7.43±1.39	12.29±1.42	13.86±2.39	14.29±1.64	8.95±1.51
p,p-DDT^4^	1.11±0.13	1.92±0.36	0.06±0.01	3.30±0.51	2.03±0.24	0.63±0.11	3.30±0.45	2.22±0.30	10.80±1.92	2.69±0.47
p,p-DDD^5^	12.59±1.95	0.46±0.07	0.27±0.05	1.27±0.21	6.22±1.00	7.29±1.26	10.13±1.76	13.29±2.25	11.23±1.52	8.43±0.87
o,p-DDT	1.32±0.23	1.33±0.14	1.95±0.22	1.43±0.20	8.70±1.01	0.79±0.12	14.16±1.86	7.08±1.03	16.08±1.67	5.55±0.74
o,p-methoxychlor	2.25±0.25	0.41±0.06	0.60±0.06	3.98±0.79	1.29±0.23	1.26±0.18	2.10±0.35	2.50±0.30	8.10±1.32	3.36±0.48
p,p-methoxychlor	1.31±0.15	0.07±0.01	0.15±0.02	0.51±0.06	0.00±0.00	0.81±0.10	< 0.05	0.93±0.15	0.91±0.18	0.81±0.09
∑PBDE 39^6^	63.02±9.27	3.98±0.54	2.45±0.44	8.50±1.23	9.19±1.80	31.84±3.23	12.61±2.11	46.73±6.32	20.29±3.96	25.27±4.62
∑PAH 28^7^	37.71±6.95	16.01±2.54	8.50±1.46	12.77±1.28	19.29±2.45	23.38±3.58	2.61±0.47	34.95±6.14	12.14±1.51	15.12±2.60
Aroclor 1260	3,890±470	113±10	80±10	189±20	270±50	2,020±280	440±70	2,940±380	660±110	1,830±240

^1^Delta isomer of hexachlorocyclohexane

^2^2-Chloro-1,1-bis(4-chlorophenyl)-ethene

^3^Sum of 2-(2-Chlorophenyl)-2-(4-chlorophenyl)-1,1-dichloroethene and 1,1-Dichloro-2,2-bis(4-chlorophenyl)ethane

^4^ 1,1,1-Trichloro-2,2-bis(4-chlorophenyl)ethane

^5^ 1,1-Dichloro-2,2-bis(4-chlorophenyl)ethane

^6^Sum of 39 evaluated polybrominated diphenyl ether congeners (see Materials and Methods section)

^7^Sum of 28 polycyclic aromatic hydrocarbons (see Materials and Methods section).

### Endocrine disrupting potency of POPs isolated from commercial fish oil

Bioassay results are given in Table 2. Calculated estradiol equivalents for all fractions eluted from the combined samples ranged from values below 0.003 pg g^–1^ up to 0.073 pg g^–1^ (fat). None of the tested samples acted as the ER antagonist. Conversely, they produced an additive response and/or show synergy and acted as ER agonists of various activity and potency in the applied assay. The alkaline fraction, i.e., the fraction potentially containing phenolic compounds [36], including metabolites of PCB/PBDE (OH-PCB/PBDE), did not show any hormonal activity in the assay and was not submitted for further evaluations. This was the most likely due to very low concentrations of these compounds in fish oil [34]. Compounds present in the dichloromethane and dichloromethane:n-hexane fractions obtained using basic alumina chromatography were responsible for the observed estrogenic potential. The hexane fraction contained octachlorostyrene, unidentified chlorinated compounds, and heptachlor. Most of the chlorinated biphenyls, DDE, trans-nonachlor, HCB and pentachlorobenzene were found in the hexane:dichloromethane fraction. DDTs, methoxychlor (DMDT), nonachlor, PBDEs and HCHs were found in the dichloromethane fraction. Relative contributions of the chemicals identified in the above fractions are shown in S1 Fig. Since the chemicals eluting in various fractions differed significantly in their spatial structure and polarity, this determined its receptor affinity and matching. In cases of substitution of isomers (like CBs congeners) slight changes in structure may significantly modify the affinity or activity of the compound [43].

**Table 2:** Calculated equivalent concentrations of (anti-)hormones for the tested oil samples. LOQ was taken as the concentration of the reference compound causing less than a 10% change in response (<EC10) assuming a 20 g sample mass.

Sample	Fraction eluted from the sample	Concentration [pg g–1]			[ng g–1]
		β-Estradiol	Tamoxifen	Dihydroxy-testosterone	Flutamide
1	All combined	0.073±0.023	< 16.3	< 2.2	216.21±42.7
	Alkaline	< 0.003			< 3.7
	Hx	< 0.003			46.07±9.7
	1% DCM	0.016±0.004			60.17±13.36
	DCM	0.017±0.006			42.16±11.19
2	All combined	0.030±0.005	< 16.3	< 2.2	120.22±33.16
	Alkaline	< 0.003			< 3.7
	Hx	< 0.003			< 3.7
	1% DCM	0.007±0.002			37.74±6.7
	DCM	0.007±0.002			35.69±11.57
3	All combined	< 0.003	< 16.3	< 2.2	46.10±13.07
	Alkaline				< 3.7
	Hx				< 3.7
	1% DCM				21.12±5.11
	DCM				11.51±2.55
4	All combined	< 0.003	< 16.3	< 2.2	44.13±9.24
	Alkaline				< 3.7
	Hx				27.86±6.57
	1% DCM				< 3.7
	DCM				< 3.7
5	All combined	< 0.003	< 16.3	< 2.2	145.07±46.1
	Alkaline				< 3.7
	Hx				10.13±2.64
	1% DCM				< 3.7
	DCM				90.05±17.78
6	All combined	0.037±0.013	< 16.3	< 2.2	79.67±13.92
	Alkaline	< 0.003			< 3.7
	Hx	< 0.003			< 3.7
	1% DCM	0.008±0.002			31.67±4.95
	DCM	0.009±0.002			22.19±6.87
7	All combined	< 0.003	< 16.3	< 2.2	18.58±6.21
	Alkaline	< 0.003			< 3.7
	Hx	< 0.003			< 3.7
	1% DCM	0.007±0.002			6.83±1.37
	DCM	0.007±0.002			4.79±0.74
8	All combined	0.054±0.012	< 16.3	< 2.2	89.15±15.2
	Alkaline	< 0.003			< 3.7
	Hx	< 0.003			< 3.7
	1% DCM	0.012±0.002			45.58±15.12
	DCM	0.013±0.003			31.94±5.86
9	All combined	0.012±0.002	< 16.3	< 2.2	23.41±3.91
	Alkaline	< 0.003			< 3.7
	Hx	< 0.003			< 3.7
	1% DCM	0.003±0.001			10.20±3.21
	DCM	0.003±9E-04			7.15±1.63
10	All combined	0.021±0.005	< 16.3	< 2.2	55.77±14.79
	Alkaline	< 0.003			< 3.7
	Hx	< 0.003			< 3.7
	1% DCM	0.008±0.002			28.52±5.82
	DCM	0.008±0.002			19.98±3.73

Hormone equivalents of the combined fractions were higher than the calculated simple sums of the equivalents of the separated fractions. This is not unusual because in most cases, hormone-dependent response curves are exponential rather than linear [26, 44–45]. The fractions were combined after chromatography to eliminate possible differences in recovery rates. Interactions between individual ligands may not be excluded at this point. Chemicals present in the fractions may also act in synergy, multiplying the observed response [46–47]. Competitiveness of ligands against ER-active sites is also possible. In addition, since the tested mixtures were rather complex, anti-hormonal activity of individual compounds may not be excluded even if such activity of the entire mixture was not indeed observed. In some cases, a subtle and specific disruption in the estrogenic signaling system of the recombinant yeast test might also occur at concentrations that would not result in apparent growth inhibition. This phenomenon has been described as toxic masking [32]. Pliskova et al. [48] reported anti-estrogenic activity of higher-chlorinated CBs while the lower chlorinated ones were weakly estrogenic. Contamination with higher-chlorinated congeners was characteristic of samples tested in this study. Lack of overall anti-estrogenic activity of the CB-containing fraction might very well be caused by some DDT metabolites or other compounds exhibiting weak estrogenic activity, acting in synergy or simply producing an additive response. Anti-estrogenic activity of Aroclor technical mixtures at the tested concentrations against E2 was not proven in this study. In contrast, an additivity or synergistic effect with E2 was observed in all Aroclor mixtures tested, see S2 Fig.

**Fig 2:**
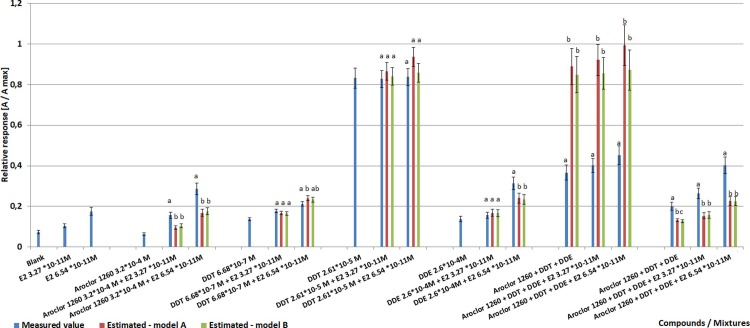
Measured and estimated relative response in the yeast estrogen assay for the tested chemicals. Aroclor 1260, DDT, and DDE concentrations in mixtures were respectively 3.2*10^−4^, 2.61*10^−5^, 2.6*10^−4^; and 4.0*10^−6^, 6.68*10^−7^, and 3.2*10^−6^ (n = 3). Statistically homologous groups have been indicated with letters (significance of the differences was tested between measured values and predictions).

To evaluate the possible interactions between the most abundant chemicals (DDT, DDE, Aroclor 1260) present in oils, some additional experiments were performed using pure standards of those chemicals and its mixtures. These results are shown in Fig 2. The experiments were performed at concentrations similar to those observed in the test samples (taking into account extract pre-concentration). The measured absorbance values for the chemical mixtures have been compared with the effects estimated from the predictive models. As noted previously, DDE, DDT and Aroclor 1260 were found to show weak estrogenicity with DDT found to be the most potent. Higher values were measured for combinations of Aroclor 1260 and E2 mixtures than that predicted from the models. Such observations were also noticed for the tested higher DDE concentrations. The results obtained with the predictive models for DDT were generally in line with the measured values, suggesting a pure additive response (at the tested concentration range). Surprisingly, the applied predicative models have failed when combined mixtures of tested chemicals were evaluated.

Hardrup et al. [49] noticed that the prediction of biological effects of hormonally active mixtures containing both chemicals with agonistic as well as chemicals having antagonistic potency with different mathematical models have significant shortcomings and do not always provide sufficient estimation. Brinkmann et al. [50] has also noted that additive models are unable to represent the effects of mixtures of partial agonists due to lesser efficacies compared to native hormones. The maximum achievable effect level and slopes of the dose-response curves are not equal for different compounds. This was also the case for the chemicals tested in our study. In addition, competitive antagonism is also possible. When the most suited model was applied, the predicted estrogenicity for the environmental samples reported by Brinkmann et al. [50] differed in the 5–622% range from the measured value. The results were both over and underestimated depending on the sample evaluated.

In addition, the course of action of the chemical mixtures tested in this study was found to be concentration dependent. At the higher (mixture) concentrations used, the measured combined effect was lower than the predicted one from the effects of individual chemicals at the tested concentrations. When E2 was added to the test mixture a higher response was observed, however not proven statistically different in case of higher mixture concentrations tested. Such observations might indicate that some of the tested chemicals showed anti-estrogenic activity in the presence of weak estrogens. This phenomenon might be explained by the higher affinity of some individual ligands than others to the receptor but not being able to activate the subsequent signaling pathway. Increased response in the presence of E2 might be explained by the extremely high affinity of native hormones to the receptor molecule. However, at lower concentrations, the same tested chemicals mixtures produced significantly higher responses that predicted from the models. This suggests synergy or at least more than additivity between the chemicals. This was also noticed when E2 was additionally added to the tested mixture. The results obtained for tested chemicals mixtures showed significant variability. The mean absorbance measured for the chemical mixture tested at a higher concentration was only proven significantly different from a mixture tested with E2 addition at 6.54*10^−11^ M. As noted previously, this might be attributed to the differences in the maximum achievable effect of individual chemicals and its receptor binding potency. Such observed alterations might be those most likely related to the chlorinated biphenyls present in the tested samples. As noted previously, they have been reported in the literature to show both estrogenic and anti-estrogenic activity [48]. According to the results of this study, such interactions are significantly concentration dependent.

On the basis of the calculated hormonal activities and determined concentrations of chemicals it may be concluded that the compounds mainly responsible for the observed disrupting potential of the isolated extracts include various DDT metabolites, PCBs, and PBDEs. o,p-DDT is the most potent estrogen among the latter compounds with 6.53 × 10^−6^ relative estrogen potency (REP) calculated from the EC50 values. Other tested compounds showed REP values within the 1 × 10^−7^–1 × 10^−9^ range (S3–S5 Figs).

### Correlation between hormone equivalents and chemical concentrations

Spearman’s rank test was used to statistically evaluate the results, see Table 3. Individual fractions were taken as cases, while hormone or anti-hormone activity and identified chemicals were taken as variables.

**Table 3:** Correlation coefficients between calculated concentration equivalents of E2 and flutamide and chemicals identified in the tested samples and fractions (Spearman rank test, α = 0.05). Statistically significant correlations are bolded.

	PeCBz	HCB	Unknown	Heptachlor	HCH-delta	Octachloro styrene	DDMU	Chlordane	Trans nonachlor	Trans chlordane
E2	0,20	**0,59**	-0,10	**0,40**	**0,49**	0,17	**0,77**	**0,70**	**0,67**	**0,63**
Flutamid	0,20	0,26	0,04	0,27	0,08	0,26	**0,42**	0,25	0,33	0,26
	DDE		Nonachlor	o,p-DDT	DDD	o,p-DDT	o,p- DMDT	p,p- DMDT	Aroclor 1260	PBDE
E2	**0,78**		**0,39**	0,26	0,15	0,12	**-0,45**	-0,10	**0,66**	**0,44**
Flutamid	**0,43**		0,07	-0,05	-0,04	0,01	-0,23	0,02	**0,38**	0,06

**Fig 3:**
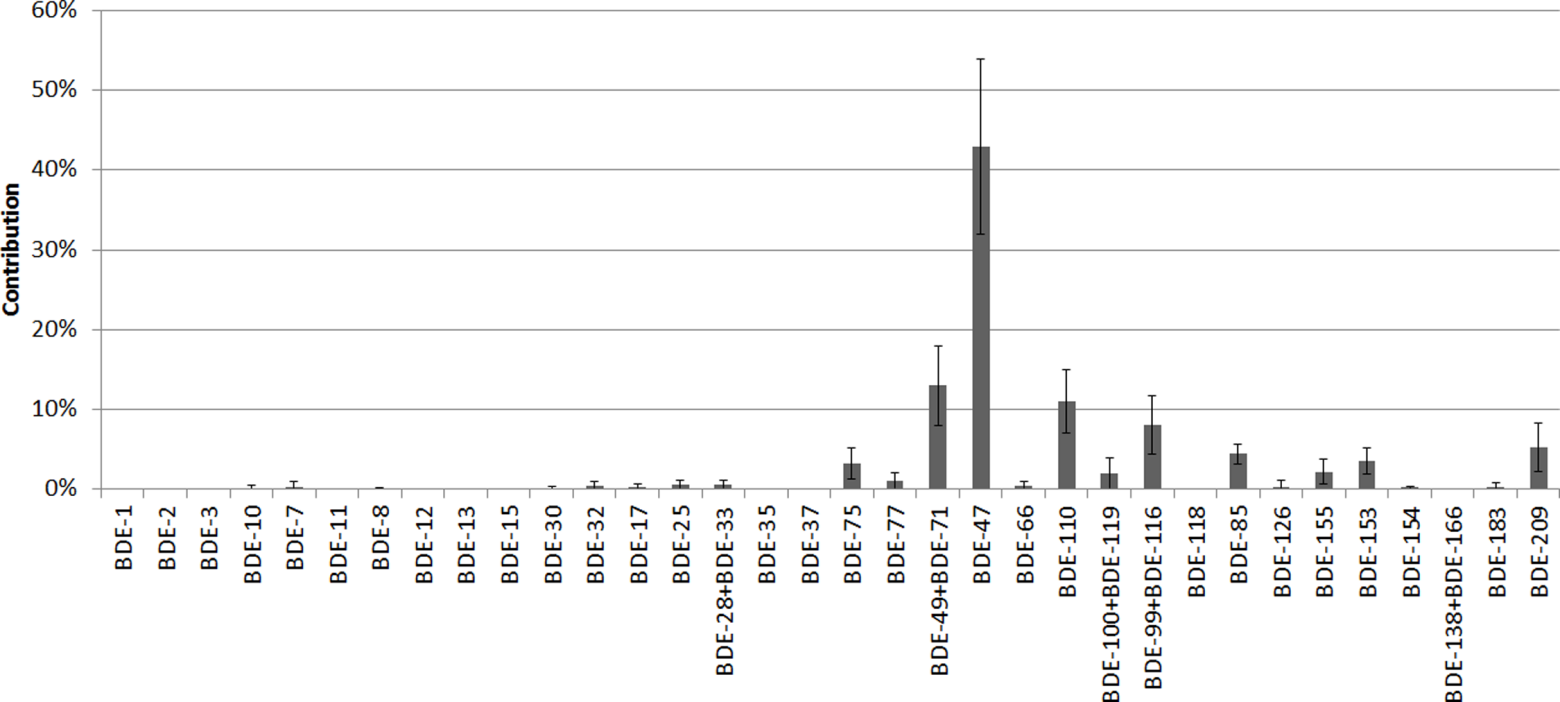
Average contribution of individual congeners to the PBDE profile in the tested fish oil samples.

It is important to note that CBs were substantively separated in the isolated fractions from PBDE/DDT metabolites except DDE. Some statistically significant monotonic correlations between concentrations and hormone equivalents are seen, including numerous correlations between the E2 equivalent and some CB/BDE/DDT metabolites. As noted earlier, that is not surprising since the latter metabolites were proven to be weak estrogens in the yeast bioassay used in this study. BDE # 47, the most conspicuous congener among all found PBDEs (see Fig 3) turned out to also be the most potent estrogen (S5 and S6 Figs). Its relatively highest share in fish oil PBDE fractions was confirmed in the literature [51–52].

Other pollutants found in the tested samples include legacy organochlorine insecticides and unidentified chlorine substituted compounds. Concentrations of some of them (e.g., cis-/trans-nonachlor) correlated statistically significantly with the calculated E2 equivalent. Hormonal activity of these pollutants were not evaluated in this study; Klotz et al. [53] reported their weak activity. We noted some HCH/E2 correlations. Additionally, Garg et al. [54] reported HCH estrogenic activity, but Kim et al. [55] reported none.

Statistical tests indicated some chlordane/E2 correlations, but Okoumassoun et al. [56] did not report any. It must be stressed that in respect to the latter compounds the indicated results and correlations may be false. Numerous POPs are frequently correlated with each other in many environmental samples. Those could not be easily isolated or fractionated on a semi-preparative scale (as applied in this study). Since DDT/PCB and HCH/chlordane coexist in the extracts at very different concentrations, it is not possible to undoubtedly evaluate their individual activity/contribution to the observed hormonal potency of the entire mixture. However, as mentioned previously, it was also assumed that no other (unidentified) compound potentially present in the tested samples was hormonally active.

The calculated estrogen equivalent was significantly correlated with the concentration of Aroclor 1260. The estrogen potency of the latter is relatively low but it is an E2 agonist (S2 Fig). This might indicate a possible additivity or synergy between chlorinated biphenyls and other weak estrogens present in food as already demonstrated previously [20,25].

All evaluated oil samples showed anti-androgenic activity in the applied assay. Estimated flutamide equivalents were in the 18.58–216.21 ng g^–1^ (fat) range. Analysis of fractionated samples indicated a significant correlation between the observed endocrine disrupting potency expressed as the flutamide concentration and the concentration of DDT metabolites and chlorinated biphenyls. In the majority of the tested samples the highest response was observed for dichloromethane and dichloromethane:hexane basic alumina fractions. The n-hexane fractions (composed of mostly aliphatic compounds) showed anti-androgenic activity in two cases (see Table 2, samples 1 and 4). However, compounds responsible for the activity were not identified (different analytical and fractionation methods would be required).

Both DDE and chlorinated biphenyls were found to show anti-androgenic activity using pure standards of those compounds (S7 and S8 Figs). These compounds have also been reported as androgen agonists [57–58]. PBDE concentration did not correlate with flutamide equivalent, most likely due to its relatively low concentration compared to CBs/DDE and its low ED potency.

### Estrogens and androgens in food

Equivalent concentrations of hormones (E2) estimated in the yeast-based bioassay are low compared to concentrations of endogenous hormones (estradiol, estrone, estriol) naturally occurring in food of animal origin. For example, cow’s milk may contain up to several hundred pg g^–1^ of endogenous estrogens, including those conjugated with proteins [59–60]. These levels are significantly higher than the equivalents calculated in this study. Animal origin food may also be a source of endogenous testosterone [59,61]. Our results are in line with Behr et al., [62] who reported low but detectable estrogenicity of farmed fish meat. The authors suggested possible residues of genistein, i.e., phytoestrogen derived from soy used as aquaculture feed. Estrogenicity of farmed fish muscle demonstrated by Pinto et al. [63] using a YES-based bioassay originated most likely (as suggested by the authors) from the presence of some POPs. Similar to the results of our study, a positive correlation between total PCB levels and the observed estrogen potency was reported by the authors.

There is not much available literature data on anti-androgens equivalents in oil samples or food. The calculated equivalent concentrations seem to be low in the tested samples with the following reservations. The studied activity referred to only one specific element of the biochemical response and only one isoform of the receptor was evaluated. In addition, every compound found in the tested samples may undergo metabolic transformation or activation in humans (including oxidation with various cytochromes), forming some more potent EDCs capable of more seriously affecting human health This has been, for example, well documented for hydroxylated metabolites of chlorinated biphenyls or hydroxylated metabolites of polycyclic aromatic hydrocarbons [18–19, 21–22, 64–67].

### Health issues

On the basis of the available literature it is difficult to unambiguously evaluate the health effects of chronic exposure to the chemicals isolated and tested in this study. Scientific communities have not reached a consensus on the potential health effects of low-dose exposure to weak EDCs [26] and consequently on the safe/tolerable exposure/intake levels. The Swedish Chemical Agency [68] identified several obstacles to this, including the complexity of the human endocrine system, variable susceptibility at various human development stages, and long time periods between exposure and occurrence of any observable adverse effects. Both the Swedish Chemical Agency, [68] and Hass et al. [44] pointed out that it was impossible to determine a threshold for a functioning system that under normal conditions was already active. In such a scenario, even the slightest change in hormone levels (hypothetically few molecules) would result in physiological effects. Endocrine disruptor activity estimations are burdened with high uncertainty, especially when experimental results are extrapolated to different species.

Dybing et al. [69] indicated that some level of interaction with or occupation of receptor active sites with endocrine disruptors must be achieved to produce toxic effects in view of the numerous mechanisms of cyto- and homoeostasis protection and the abundance of cellular and molecular elements able to interact with hormonal signaling. Body homeostasis mechanisms are able to counteract any disrupting action produced by xenobiotics at levels below the critical thresholds. However, if the mechanisms are less operational at some stage of organism development, susceptibility to xenobiotics appearing at that stage might be higher.

Regardless of the above, the results of estrogen equivalents calculated for the tested samples may be compared with the acceptable daily intake (ADI) values reported in the literature for the reference compounds. Such an approach has been previously adopted by Caldwell et al. [70]. The WHO [71] proposed an ADI of 0.05 μg E2 kg^-1^ b.w. day^-1^ based on a no observed effect level (NOEL). For the purpose of comparison of fish oil related exposure to the toxicity-based benchmark, a conservative scenario was adopted including the following assumptions. An average adult body weight of 60 kg, a maximum concentration (0.073 pg g^-^1) was used for calculations, and 80 g dietary fat intake (solely from fish oil). Based on such assumptions the average calculated E2 equivalent would amount to 5.84 pg E2 equivalent person^-1^ day^-1^. This number is much lower than the 3 μg E2 equivalent person^-1^ day^-1^ ADI value. Using the same assumptions, the intake of flutamide equivalent was estimated at 17.28 μg person^-1^ day^-1^. The ADI value for flutamide not causing anti-androgenic effects was set at 2.5 μg kg^-1^ b.w. day^-1^ which results in 150 μg kg^-1^ person^-1^ day^-1^ [72]. The estimated values in the worst-case scenario are only 10 times lower than the ADI. Despite the fact that the adopted worst-case scenario is unlikely, the calculated intake value might suggest that extensive use of contaminated fish oil in some (sensitive) population groups might be a health concern if anti-androgenic effects are evaluated. In addition, as noted by De Falca et al. [73], a balance between androgens and estrogens may be important in maintaining normal spermatogenesis. Since the tested oil samples showed both estrogenic and anti-androgenic activity, its biological activity might be significantly stronger due to its complex mechanism of action. Exposure to xenoestrogens and anti-androgens during fetal and neonatal development has already been associated with a series of male reproductive disturbances, such as cryptorchidism, hypospadias, impaired fertility (especially due to poor semen quality), and an increased incidence of testicular cancer [73].

As noted previously, limitations of the model and tests applied in this study should be strongly considered. In addition, the mode of action of the chemicals evaluated is only one element in its toxicological profile, which includes carcinogenicity, genotoxicity or interactions with other NRs (including aryl hydrocarbon). This indicates that such contaminated fish oils might produce undesirable health effects, including sex hormone system disruption, despite providing valuable nutrients if used as a food.

## Conclusions

The results of this study confirm that residues of POPs present in fish oil may disrupt the human endocrine system by means of agonistic and antagonistic effects on human estrogen and androgen receptors. Equivalent concentrations of the native hormone (E2) estimated using a yeast-based bioassay were low as compared to concentrations of endogenous hormones (estradiol, estrone, estriol) naturally occurring in food of animal origin. However, every tested sample showed some measurable anti-androgenic activity. Residues of polychlorinated biphenyls, DDT, and its metabolites contributed the most to the endocrine disrupting potency in comparison with other POPs present in our fish oil samples.

## Supporting information

S1 Fig Relative contribution of chemicals identified in the basic alumina chromatography fractions. (DOCX)

S2 Fig Relative response in yeast estrogen assay for the tested Aroclor mixtures in the presence of E2 at 2 x 10^−10^ M (n = 3). Control–first bar blank, second bar blank + E2. Maximum response from the E2 dose-response curve was used for calculations as a reference maximum effect (Amax).
(DOCX)

S3 Fig Dose-response curves for the tested Aroclor mixtures against ER (n = 3). (DOCX)

S4 Fig Dose-response curves for the tested polybrominated biphenyls ethers against ER (n = 3). (DOCX)

S5 Fig Relative response in yeast estrogen assay for the tested PBDEs in the presence of E2 at 2 x 10^−10^ M (n = 3). Control–first bar blank, second bar blank + E2.
(DOCX)

S6 Fig Dose-response curves for the o,p-DDT and p,p-DDE. OH-tamoxifen (HT) was used as an anti-estrogen positive control (n = 3).
(DOCX)

S7 Fig Antagonist response curves for DDE and Aroclor 1232 against AR. Flutamide was used as anti-androgen positive control (DHT concentration at 2,78 x 10^−9^ M). DHT was plotted as agonist positive standard (n = 3).
(DOCX)

S8 Fig Antagonist response curves for BDE #47, 99, 100 against AR. Flutamide was used as anti-androgen positive control (DHT concentration at 2,78 x 10^−9^ M) (n = 3). DHT was plotted as agonist positive standard.
(DOCX)

S1 Table Limits of quantification, limits of determination, recovery rates and recovery relative standard deviation for the evaluated organochlorine insecticides and related compounds. N/D–not determined.
(DOCX)
