# Supplementary material for: Endocrine disrupting potency of organic pollutant mixtures isolated from commercial fish oil evaluated in yeast-based bioassays
Source: PLoS One. 2018 May 22;13(5):e0197907. doi: 10.1371/journal.pone.0197907 (PMC5963795; doi:10.1371/journal.pone.0197907)
Supplement: S1 Fig — (DOCX) [file pone.0197907.s001.docx]

*
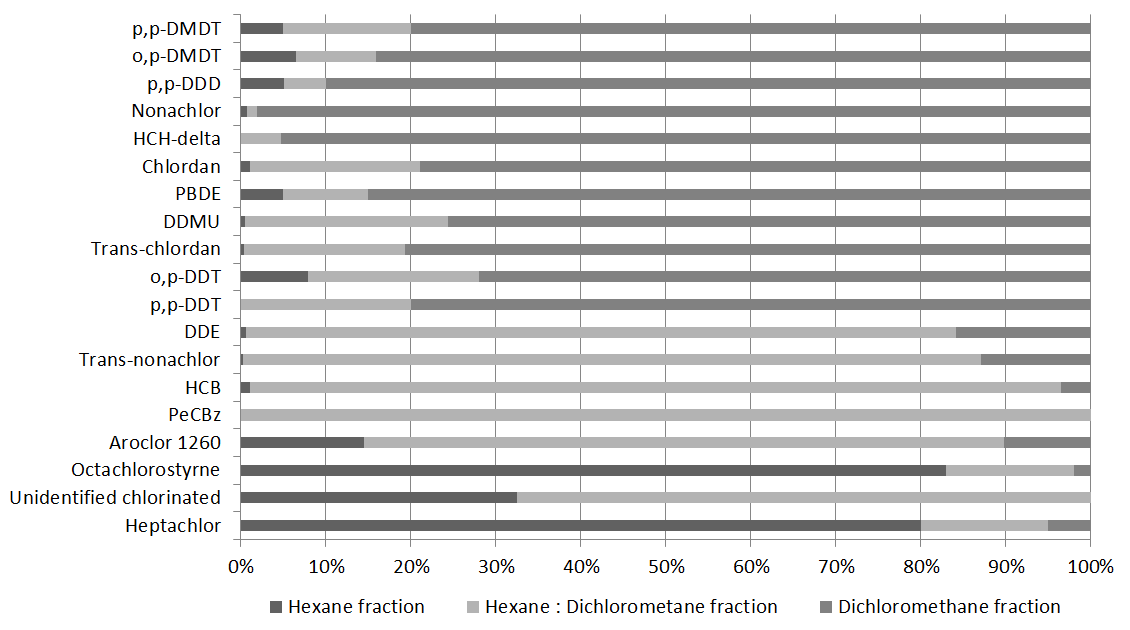
*

**S1 Fig. Relative contribution of chemicals identified in the basic alumina chromatography fractions**
