# Supplementary material for: Endocrine disrupting potency of organic pollutant mixtures isolated from commercial fish oil evaluated in yeast-based bioassays
Source: PLoS One. 2018 May 22;13(5):e0197907. doi: 10.1371/journal.pone.0197907 (PMC5963795; doi:10.1371/journal.pone.0197907)
Supplement: S2 Fig — Control–first bar blank, second bar blank + E2. Maximum response from the E2 dose-response curve was used for calculations as a reference maximum effect (Amax). (DOCX) [file pone.0197907.s002.docx]

*
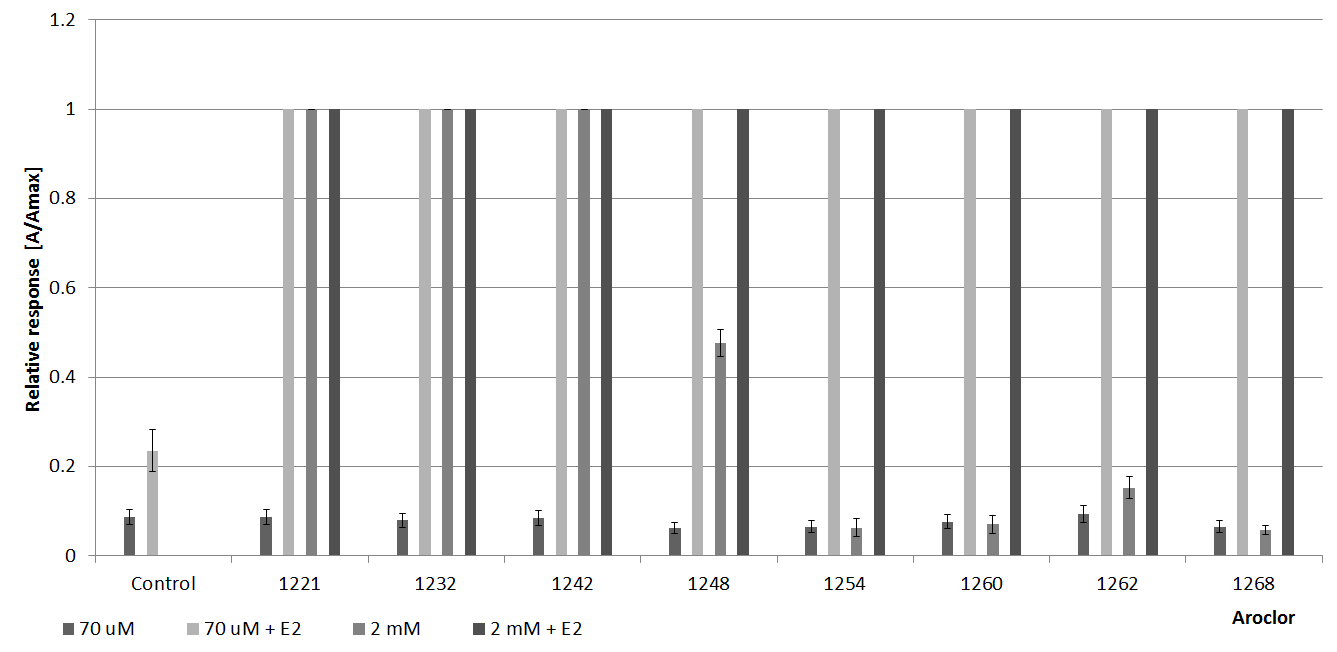
*

**S2 Fig. Relative response in yeast estrogen assay for the tested Aroclor mixtures in the presence of E2 at 2 x 10^–10^ M (n=3).** Control –first bar blank, second bar blank + E2. Maximum response from the E2 dose-response curve was used for calculations as a reference maximum effect (Amax).
