# Supplementary figures and images for: Endocrine disrupting potency of organic pollutant mixtures isolated from commercial fish oil evaluated in yeast-based bioassays
Source: PLoS One. 2018 May 22;13(5):e0197907. doi: 10.1371/journal.pone.0197907 (PMC5963795; doi:10.1371/journal.pone.0197907)

*
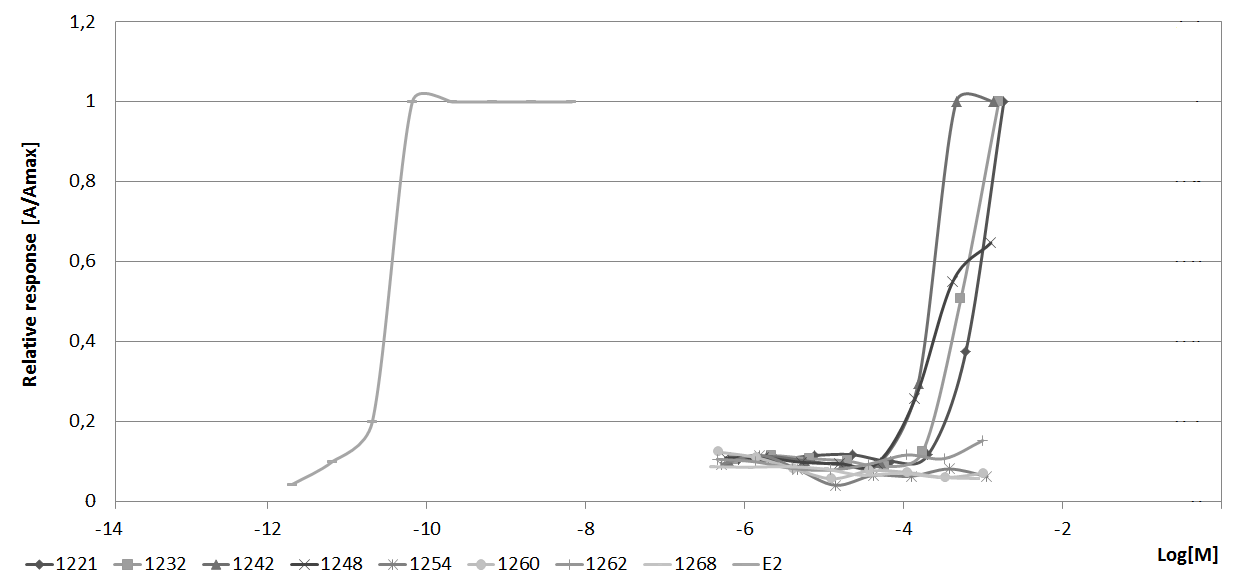
*

**S3 Fig. Dose-response curves for the tested Aroclor mixtures against ER (n=3)**

Supplement: S3 Fig — (DOCX) [file pone.0197907.s003.docx]

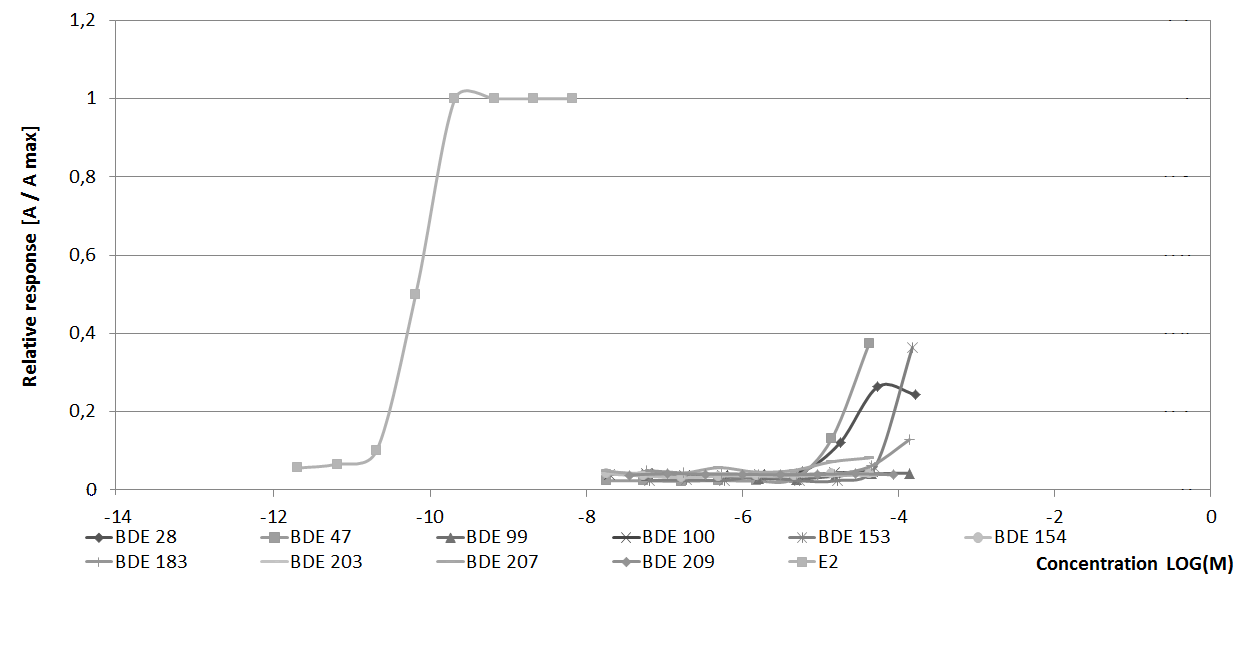


**S4 Fig. Dose-response curves for the tested polybrominated biphenyls ethers against ER (n=3)**

Supplement: S4 Fig — (DOCX) [file pone.0197907.s004.docx]
