# Supplementary material for: Endocrine disrupting potency of organic pollutant mixtures isolated from commercial fish oil evaluated in yeast-based bioassays
Source: PLoS One. 2018 May 22;13(5):e0197907. doi: 10.1371/journal.pone.0197907 (PMC5963795; doi:10.1371/journal.pone.0197907)
Supplement: S5 Fig — Control–first bar blank, second bar blank + E2. (DOCX) [file pone.0197907.s005.docx]

*
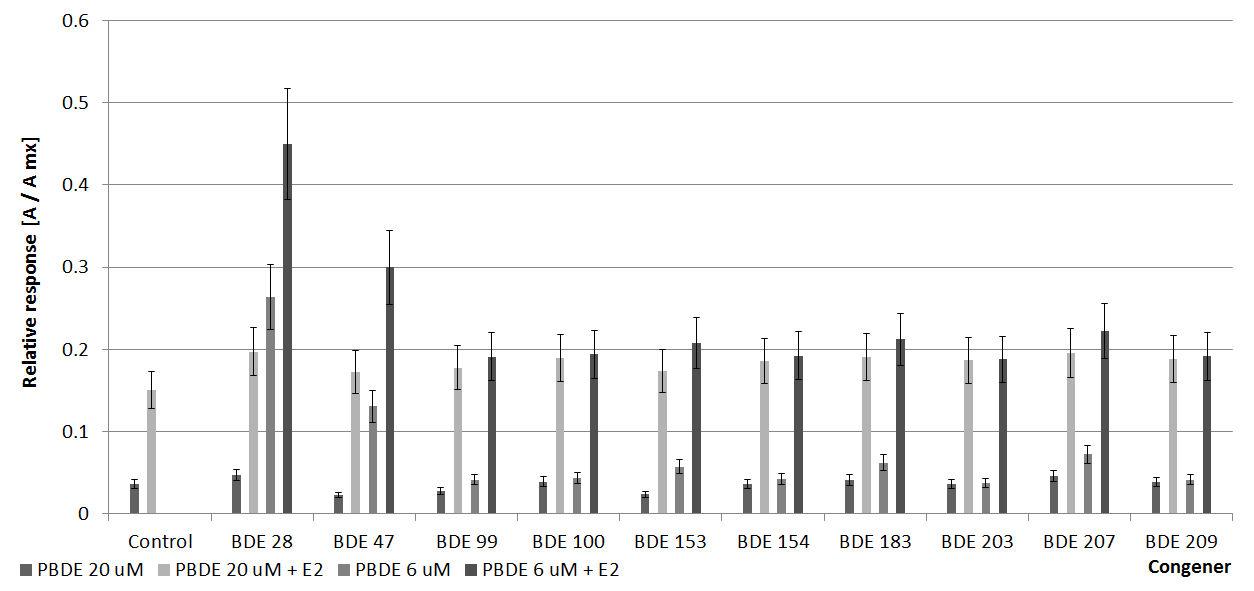
*

**S5 Fig. Relative response in yeast estrogen assay for the tested PBDEs in the presence of E2 at 2 x 10^–10^ M (n=3).** Control – first bar blank, second bar blank + E2.
