# Supplementary material for: Endocrine disrupting potency of organic pollutant mixtures isolated from commercial fish oil evaluated in yeast-based bioassays
Source: PLoS One. 2018 May 22;13(5):e0197907. doi: 10.1371/journal.pone.0197907 (PMC5963795; doi:10.1371/journal.pone.0197907)
Supplement: S6 Fig — OH-tamoxifen (HT) was used as an anti-estrogen positive control (n = 3). (DOCX) [file pone.0197907.s006.docx]

*
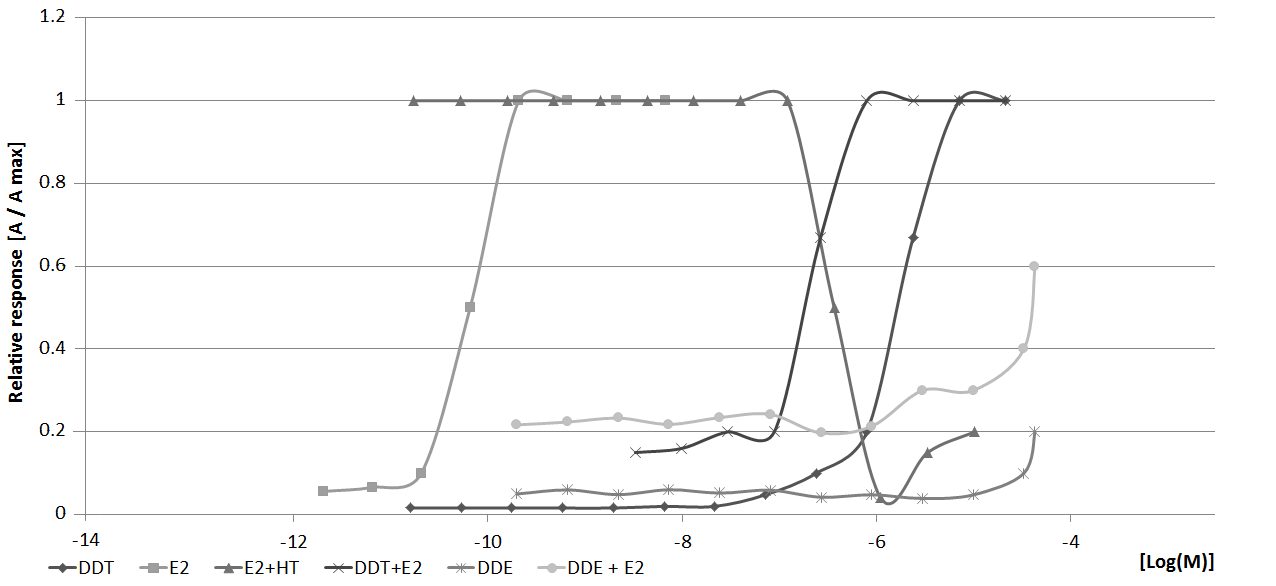
*

**S6 Fig. Dose-response curves for the o,p-DDT and p,p-DDE. OH-tamoxifen (HT) was used as an anti-estrogen positive control (n=3).**
