# Supplementary material for: Endocrine disrupting potency of organic pollutant mixtures isolated from commercial fish oil evaluated in yeast-based bioassays
Source: PLoS One. 2018 May 22;13(5):e0197907. doi: 10.1371/journal.pone.0197907 (PMC5963795; doi:10.1371/journal.pone.0197907)
Supplement: S7 Fig — Flutamide was used as anti-androgen positive control (DHT concentration at 2,78 x 10^−9^ M). DHT was plotted as agonist positive standard (n = 3). (DOCX) [file pone.0197907.s007.docx]

*
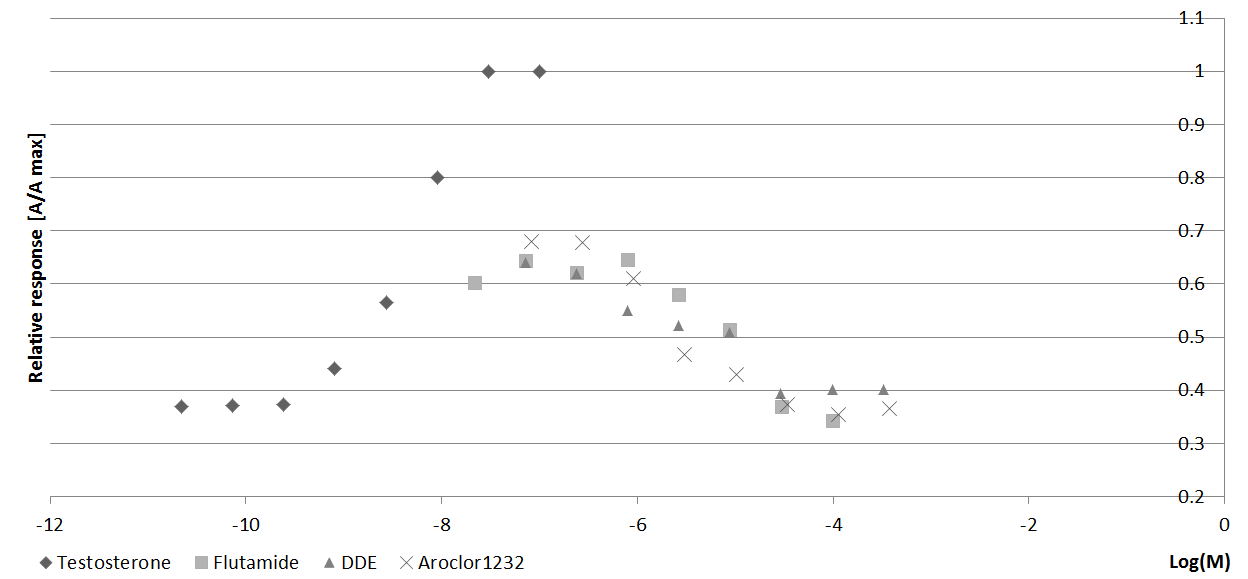
*

**S7 Fig. Antagonist response curves for DDE and Aroclor 1232 against AR. Flutamide was used as anti-androgen positive control (DHT concentration at 2,78 x 10^-9^ M).** DHT was plotted as agonist positive standard (n=3).
