# Supplementary material for: Endocrine disrupting potency of organic pollutant mixtures isolated from commercial fish oil evaluated in yeast-based bioassays
Source: PLoS One. 2018 May 22;13(5):e0197907. doi: 10.1371/journal.pone.0197907 (PMC5963795; doi:10.1371/journal.pone.0197907)
Supplement: S8 Fig — Flutamide was used as anti-androgen positive control (DHT concentration at 2,78 x 10^−9^ M) (n = 3). DHT was plotted as agonist positive standard. (DOCX) [file pone.0197907.s008.docx]

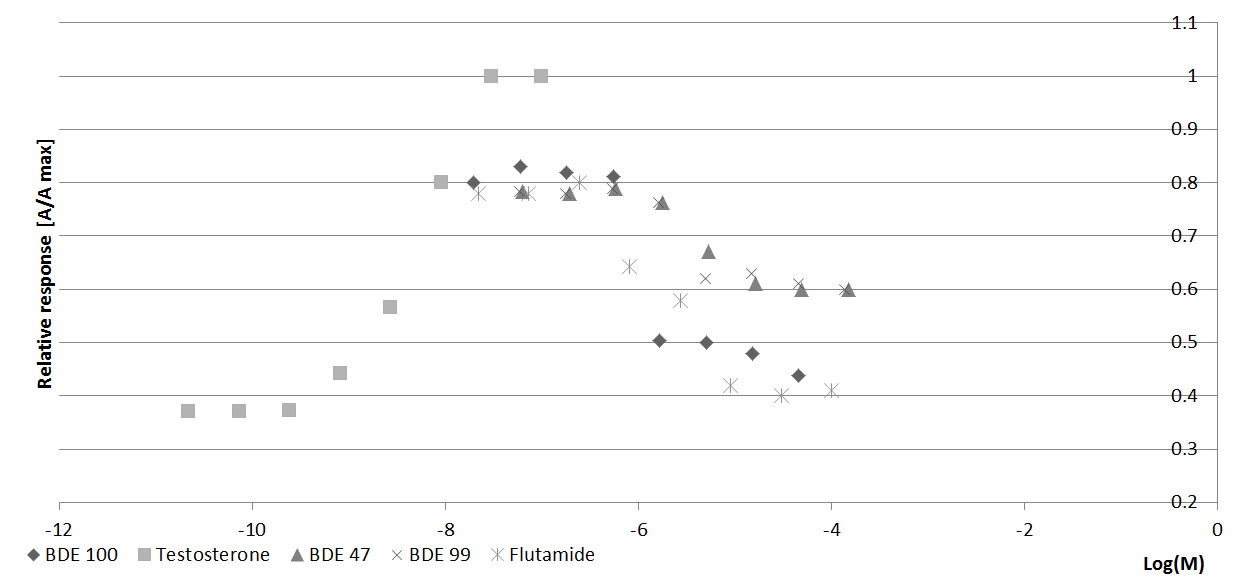


**S8 Fig. Antagonist response curves for BDE #47, 99, 100 against AR. Flutamide was used as anti-androgen positive control (DHT concentration at 2,78 x 10^-9^ M) (n=3).** DHT was plotted as agonist positive standard.
