# Supplementary material for: Endocrine disrupting potency of organic pollutant mixtures isolated from commercial fish oil evaluated in yeast-based bioassays
Source: PLoS One. 2018 May 22;13(5):e0197907. doi: 10.1371/journal.pone.0197907 (PMC5963795; doi:10.1371/journal.pone.0197907)
Supplement: S1 Table — N/D–not determined. (DOCX) [file pone.0197907.s009.docx]

**S1 Table. Limits of quantification, limits of determination, recovery rates and recovery relative standard deviation for the evaluated organochlorine insecticides and related compounds.** N/D – not determined.

| Compound | LOD | LOQ | Recovery | RSD_R_ |
| --- | --- | --- | --- | --- |
|  | ng g^-1^ | | (n=6) | |
| PeCBz | 0,002 | 0,01 | N/D | |
| HCB | 0,006 | 0,02 | 83% | 13% |
| Unknown chlorinated | 0,450 | 1,50 | N/D | |
| Heptachlor | 0,015 | 0,05 | 80% | 8% |
| HCH-delta | 0,015 | 0,05 | 76% | 10% |
| Octachlorostyrene | 0,060 | 0,20 | N/D | |
| DDMU | 0,300 | 1,00 | 79% | 14% |
| Chlordane | 0,060 | 0,20 | 80% | 11% |
| Trans-nonachlor | 0,030 | 0,10 | N/D | |
| Trans chlordane | 0,030 | 0,10 | 82% | 11% |
| DDE | 0,300 | 1,00 | 82% | 13% |
| Nonachlor | 0,016 | 0,05 | N/D | |
| p,p-DDT | 0,015 | 0,05 | 77% | 11% |
| p,p-DDD | 0,030 | 0,10 | 90% | 15% |
| o,p-DDT | 0,030 | 0,10 | 88% | 16% |
| o,p-DMDT | 0,015 | 0,05 | 79% | 8% |
| p,p-DMDT | 0,015 | 0,05 | 79% | 12% |
